# Arbitrariness of bibliometric parameters: a case study on leading scientists in the German Society for Experimental and Clinical Pharmacology and Toxicology (DGPT)

**DOI:** 10.1007/s00210-024-03195-4

**Published:** 2024-06-12

**Authors:** Louisa Christin Fox, Roland Seifert

**Affiliations:** Institute of Pharmacology, Hannover Medical School, Carl-Neuberg-Str. 1, D-30625 Hannover, Germany

**Keywords:** Bibliometric comparison, Scientific productivity, Gender research, Elite research, Pharmacology, DGPT

## Abstract

Bibliometric rankings of researchers are increasingly important for academic hiring and for making grant application decisions in the biomedical sciences. As a case study, we performed a comprehensive bibliometric analysis of German pharmacology and toxicology. The 42 members of the German Society for Experimental and Clinical Pharmacology and Toxicology (DGPT) represented in the German ‘best scientist’ ranking in biology and biochemistry on www.research.com for the year 2022 were analyzed according to various aspects. The scientist ranking on *Research.com* is based on the Hirsch Index (h-Index). In the comparatively small field of pharmacology, which accounts for only 4.2% of the scientists in the ranking on *Research.com*, there are only two women. This shows that female pharmacologists are highly underrepresented in elite pharmacology. To achieve a high h-Index, a pharmacologist must publish more papers than a biochemist or biologist. Furthermore, German elite pharmacology was compared in the three sub-societies of the DGPT. There are no significant differences between elite pharmacologists and toxicologists in terms of productivity. Two large German pharmacology schools (Günter Schultz and Franz Hofmann) are similar in all bibliometric parameters except for number of total publications. Age-specific factors were also defined for the analysis: ‘academic age’ and the quotient of the h-Index by ‘academic age’. Any given bibliometric parameter (or combination of parameters) yielded different ranking results. This became even more evident when additionally considering the highly popular and widely used *Laborjournal* ranking of top pharmacology and toxicology researchers with only very few DGPT members listed. We unmasked 7 types of publication patterns of pharmacologists, an age-dependent publication peak at around 55 years and different trajectories for high- and low-volume publishing pharmacologists. In the future, less emphasis should be paid to bibliometric parameters in academic hiring and grant decisions than to the authentic societal and scientific impact of the research. Bibliometric parameters are very arbitrary within a very large segment of pharmacologists. Studies according to the paradigm of this account should be made for other countries, other learned societies, and other scientific fields. The different cultures among related scientific fields must be considered in bibliometric analyses as exemplified here for pharmacology *versus* biochemistry. Conversely, the bibliometric similarities between pharmacology and toxicology show that both fields belong together and have a very similar culture.

## Introduction

There are large differences in the performance of scientists in the different scientific disciplines when analyzed using bibliometric analysis parameters. This is particularly noticeable in the heterogeneous group of biologists and biochemists in the ‘best scientist’ ranking on *Research.com*. Pharmacology forms a rather small part of these scientists. The size of the research field and factors such as horizontal and vertical segregation contribute to the fact that the proportion of women in the cohort of elite researchers is low (Alonso et al. 2016; Luukkonen-Gronow 1987; Mabandla 2001). Worldwide, the proportion of women in research and development was 29.3% in 2016 (UNESCO Institute for Statistics 2019). To evaluate the scientific performance of researchers, the present work will be based on various partly new defined bibliometric analysis parameters, which are summarized in Table 1.

**Table 1:** Bibliometric parameters, each with meaning and limitations

Parameter	Meaning	Limitations
Citations	*Total number of citations of a researcher* Possible measure of scientific perception and recognition in the scientific community	-Comparability between disciplines -Not very specific, merely an added-up, generalized value -Self-citations and alliances of scientists who frequently quote each other
Publications	*Total number of publications of a researcher* Possible measure of scientific productivity	-No information on the value of publications and their quality -Breakdown of publications into individual articles to increase the number
h-Index (Hirsch Index)	*Bibliometric index defined by Jorge E. Hirsch: number of papers h with citation number of ≥ h *(Hirsch 2005) Possible measure of the scientific recognition of a researcher. Exclusion of the ‘extremes’, very much and very little cited publications do not have such a strong impact (Bornmann et al. 2008). Is based on a list of publications in descending order of citations, considering both the actual scientific productivity in form of publications and the impact of a scientist measured by citations. Explanation: A researcher who has an h-Index of 10 must have published at least 10 publications and each of these 10 publications must have been cited at least 10 times	-Comparability between disciplines (popularity and size of the discipline) -Comparability between different publication types -Can also increase after death through further citations (but limited by the researcher’s publications no longer increasing) and cannot decrease
C/P-Index	*Quotient of citations by publications *(Dashun and Barabási 2021) Possible measure of the average scientific recognition of a researcher. Indicates how many citations a researcher receives on average per publication $\mathrm C/\mathrm P-\mathrm{Index}=\frac{\mathrm{Citations}}{\mathrm{Publications}}$	-Index does not require correction mechanisms and can be strongly boosted by a few publications that have received a high number of citations
h/P-Index	*Quotient of h-Index by publications* Possible measure of the scientific consistency of a researcher. As a quotient of the h-Index and publications, a relative indication is calculated of how many publications achieve such high citations that they can support the h-Index. If a researcher achieves a consistently high number of citations for their publications, their h/P-Index is closer to 1 $\mathrm h/\mathrm P-\mathrm{Index}=\frac{\mathrm h-\mathrm{Index}}{\mathrm{Publications}}$	-Orientation to prior bibliometric parameters and no full elimination of stated problems -Possible distortion in the areas of extremely high publication numbers
‘academic age’	*Age of the scientist minus 25 years* Possible measure for a better understanding of the duration of a scientist’s academic career in order to make age-specific comparisons. Since an academic career after graduation does not usually begin until the age of 25, this calculation is intended to more accurately analyze the actually important period and duration of a researcher’s academic productivity $\mathrm{academic}\;\mathrm{age}=\mathrm{age}-25\;\left(\mathrm{in}\;\mathrm{years}\right)$	-Generalization of biographies: not every (elite) scientist begins their academic career according to the same pattern directly after graduating from school and university -Individual careers or barriers as well as family planning can distort the parameter (especially for women)
h-Index/‘academic age’	*Quotient of h-Index by ‘academic age’* Possible measure for an ‘academic age’-adjusted h-Index. Attempt to analyze the h-Index in terms of the corresponding duration of the academic career. The division by ‘academic age’ is intended to make it easier to compare high h-Indices resulting from a long academic career (see limitations of the h-Index; it can also increase for life after the end of an academic career or even posthumously) $\mathrm h-\mathrm{Index}/'\mathrm{academic}\;\mathrm{age}'=\frac{\mathrm h-\mathrm{Index}}{'\mathrm{academic}\;\mathrm{age}'}$	-No complete elimination of the disadvantages of the h-Index
Publications per year (PPY)	*Number of publications by a scientist in a calendar year* Possible measure for the individual scientific productivity of a scientist in the form of publications. Illustrates the different and unique scientific careers of a researcher by showing fluctuations in publications per year	-Does not take into account the variable reasons for fluctuating publications or scientific activity, e.g., shifted professional activities or personal reasons -Outliers are more prominent than in the 2-year period
Publications per 2 years (PP2Y)	*Added number of publications by a scientist in 2 calendar years* Possible measure for the individual scientific productivity of a scientist in the form of publications. Intended to compensate for strong outliers in publications per year	-See PPY above

The publicly available ‘best scientist’ ranking on *Research.com* for the year 2022 is the source for the extracted and analyzed data. It is centered around the Hirsch Index (h-Index; see Table 1) (Hirsch 2005). Researchers in various disciplines with an h-Index of 40 or more are ranked according to this parameter. Additional information consists of the number of citations, publications and the research location. Several members of the German Society for Experimental and Clinical Pharmacology and Toxicology (DGPT) can also be assigned to the group of biologists and biochemists in the German ‘best scientist’ ranking in this field.

The DGPT consists of a total of 2414 members (March 2024) in 3 sub-societies: the Society for Toxicology (GT), the German Society for Pharmacology (DGP), and the German Society for Clinical Pharmacology and Therapy (DGKliPha). The GT is the largest sub-society of the DGPT. In the European Union, each chemical company needs to have its own board-certified toxicologist by law (https://toxikologie.de/weiterbildung/weiterbildungsprogramm-fachtoxikologe-gt/; https://www.eurotox.com). Therefore, becoming a toxicologist is very attractive for having a successful career in industry. This bolsters the membership of the GT very substantially. In contrast, there is no German or European Union legal requirement for industry pharmacologists to be board-certified, reducing the incentive to become member of the DGP or DGKliPha.

**Fig. 1:**
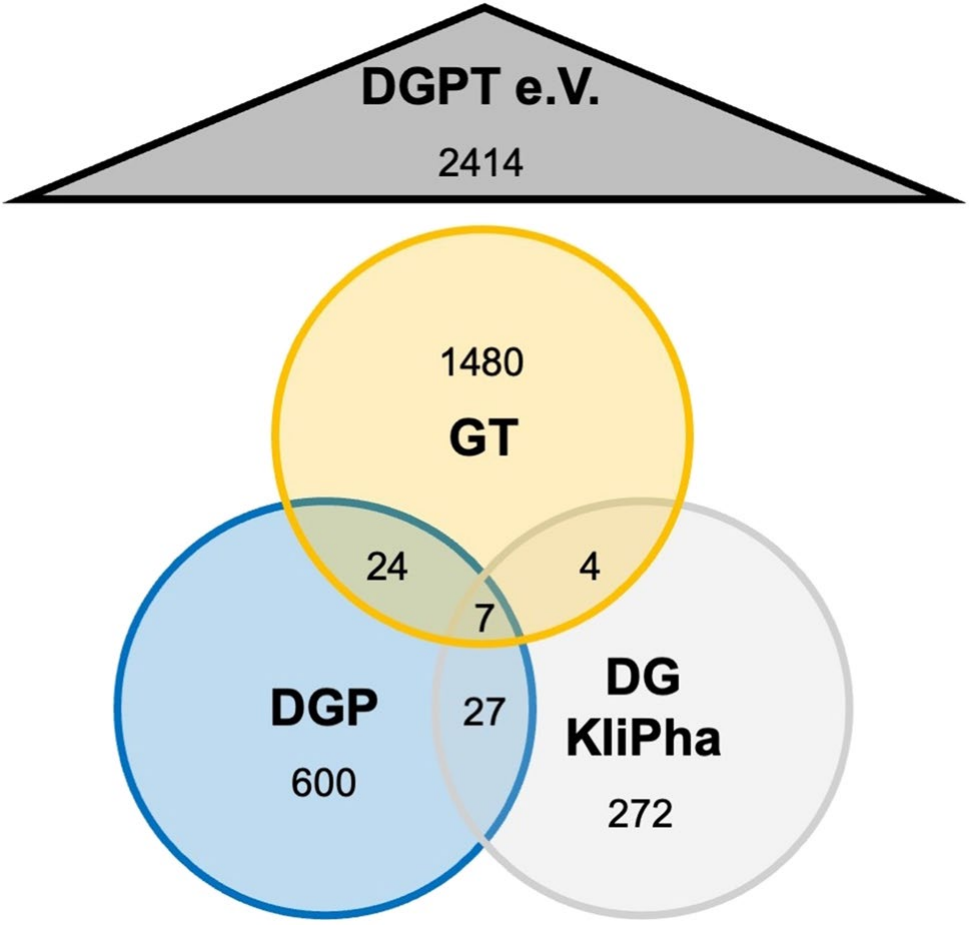
Structure of the DGPT with its three sub-societies. The double and triple memberships are taken into account in the overlapping areas of the circles. In total, the DGPT consists of 2414 individual members. The data is valid as of 1st March 2024 as was kindly provided by the DGP. e.V. = eingetragener Verein (registered association)

Figure 1 illustrates the structure of the DGPT with its 3 sub-societies and the membership counts. The goal of the DGPT is to ‘promote the scientific and practical interests of pharmacology and toxicology’ (DGPT 2024). The DGPT is particularly suitable for this type of analysis because further sub-analyses can be carried out: both the sub-societies that make up the DGPT and the ‘pharmacological schools’ can be compared with each other. In this case, the pharmacological schools of Günter Schultz and Franz Hofmann were analyzed in more detail.

This analysis therefore aims to answer the following research questions:

1. How does elite German pharmacology and toxicology as a discipline compare to the rest of the biologists and biochemists in the *Research.com* ranking in terms of bibliometric and gender issues?
2. Are there significant differences between the major pharmacological schools in Germany or the sub-societies of the DGPT?
3. How comparable and fair are bibliometric analyses that are possibly cross-disciplinary and related to only one parameter? The latter question is particularly important in light of the fact that nowadays many academic hiring and grant application decisions are strongly bibliometric parameter-driven. H-Indices, number of publications and citations are used as surrogate parameters for scientific quality and creativity, resulting in misuse (Ioannidis and Maniadis 2024).

## Materials and methods

**Fig. 2:**
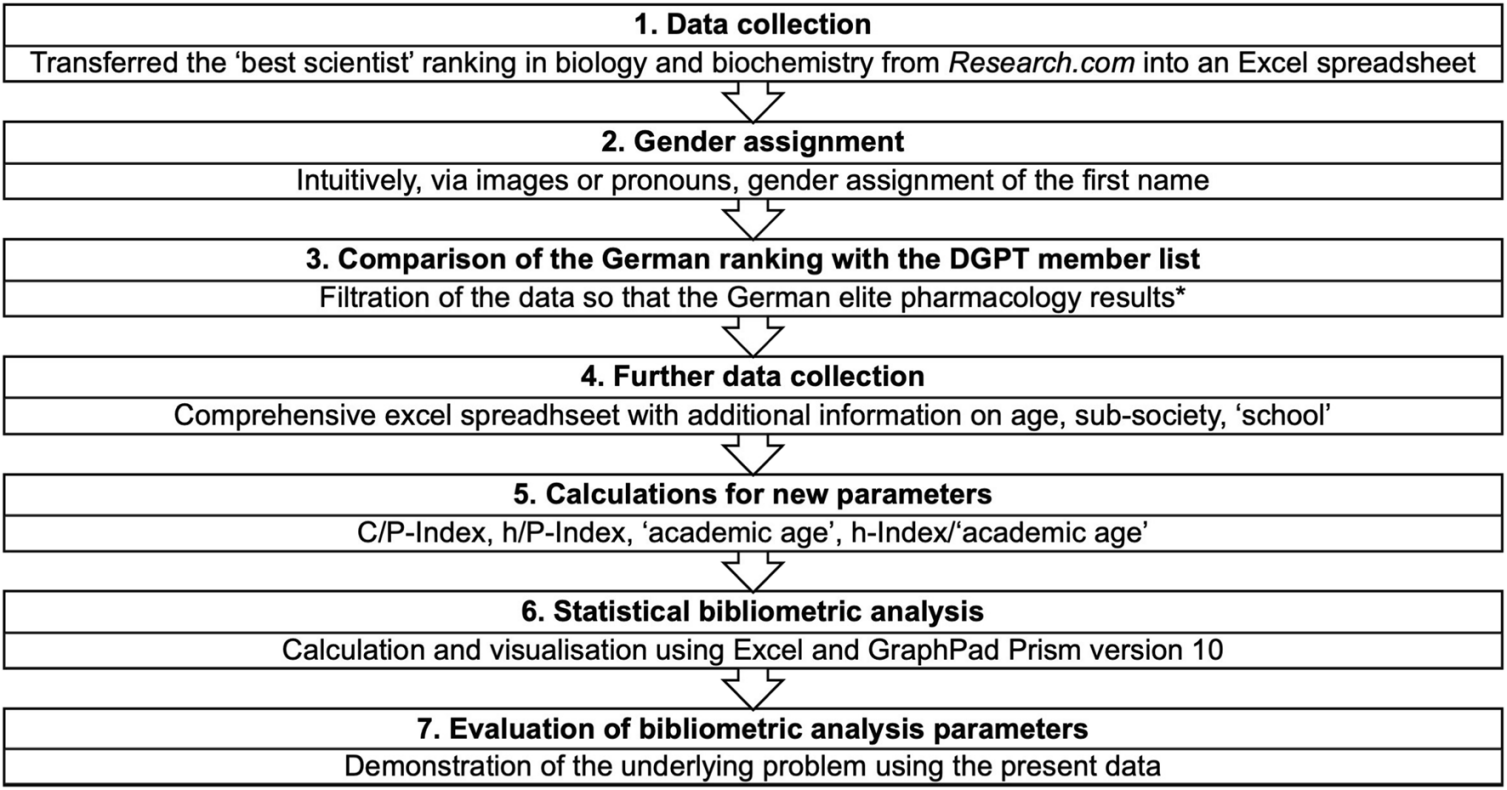
Illustration of the methodical process of this bibliometric analysis. Marked with an asterisk is an important limitation in the cohort selection: the assignment of scientists by *Research.com* to the corresponding fields leads to the fact that some members of the DGPT are represented in ‘best scientist’ rankings, but were not classified in biology and biochemistry, but in medicine, for example. They are therefore not represented in the data set. This must be taken into account in the definition of the group

The methodical procedure is illustrated in Fig. 2. The study focused on the elite scientists of the field of biology and biochemistry and was broken down further (Table 2). The German ‘best scientist’ ranking on *Research.com* (year 2022) from the field of biology and biochemistry, to which pharmacology can also be largely assigned, was compared with the DGPT member list. The member list was kindly supplied for analysis purposes by the DGPT administrative office and transferred to a new Excel spreadsheet. It was defined that a scientist is only a pharmacologist or toxicologist if they are a member of one of the sub-societies of the DGPT, reflecting the commitment of the researcher to support the development of the discipline. This is an unambiguous professional definition. For the 42 matches, the information on national rank, world rank, name, institution, h-Index, citations, publications, h/P-Index, C/P-Index, gender and membership in the sub-societies was then provided. The table was completed with further information: the age of the researchers and their affiliation to various pharmacological ‘schools’. These data were used to conduct a comprehensive bibliometric, gender and age analysis of German elite pharmacology, which was also compared with previous studies (Bünemann and Seifert 2024; Zehetbauer et al. 2022; Zöllner and Seifert 2023). In addition, the group of pharmacologists was compared in various parameters with the group of other biologists/biochemists from the scientist ranking.

**Table 2:** Overview of the analyzed data. The sample sizes are given including the percentage of the specializations from the main group biology and biochemistry. Data from Germany tabulated on *Research.com* were analyzed

	Biology and biochemistry (*N* = 999; 100%)	Other specializations (*N* = 957; 95.8%)	Pharmacology (*N* = 42; 4.2%)
Male (%)	880 (88.1%)	840 (87.8%)	40 (95.2%)
Female (%)	119 (11.9%)	117 (12.2%)	2 (4.8%)

The more detailed statistical analysis and visualization was carried out using GraphPad Prism version 10 by correlating various parameters with each other and establishing relationships. In addition, the bibliometric parameters were tested for significance by using the Mann-Whitney test, because the data was not normally distributed. A two-way ANOVA was used to compare the ‘pharmacological schools’.

Finally, the bibliometric analysis parameters were visualized using a heat map in Excel to illustrate the significance and reliability of the parameters and to clarify a major ‘problem’ of bibliometric analyses—the comparability.

## Results and discussion

### Women in pharmacology

**Fig. 3:**
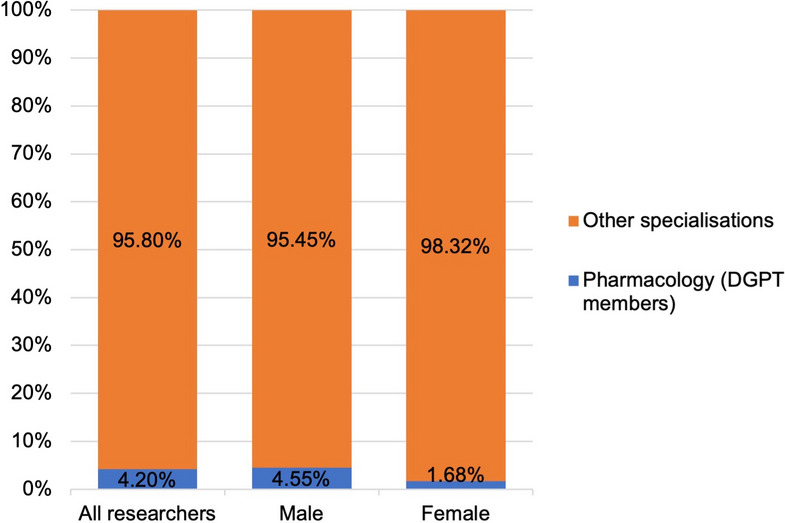
Proportion of pharmacologists among the German ‘best scientists’ in the field of biology and biochemistry on *Research.com*, listed separately for the genders

Figure 3 shows the percentage composition of the data set analyzed. Of the German ‘best scientists’ (*Research.com*, year 2022) in the field of biology and biochemistry, 4.2% are members of the DGPT (42 researchers in total). The proportion of men in pharmacology is much higher than that of women: in the analyzed main category of biology and biochemistry, only 1.68% of women in Germany can be assigned to the field of pharmacology. It should be noted that not all scientists working in the field of pharmacology are members of the DGPT and not all elite scientists in the DGPT are assigned to the biology and biochemistry category in the *Research.com* ranking. The premise and limitation of this study is therefore that the group of DGPT members analyzed represents German (elite) pharmacology.

**Fig. 4:**
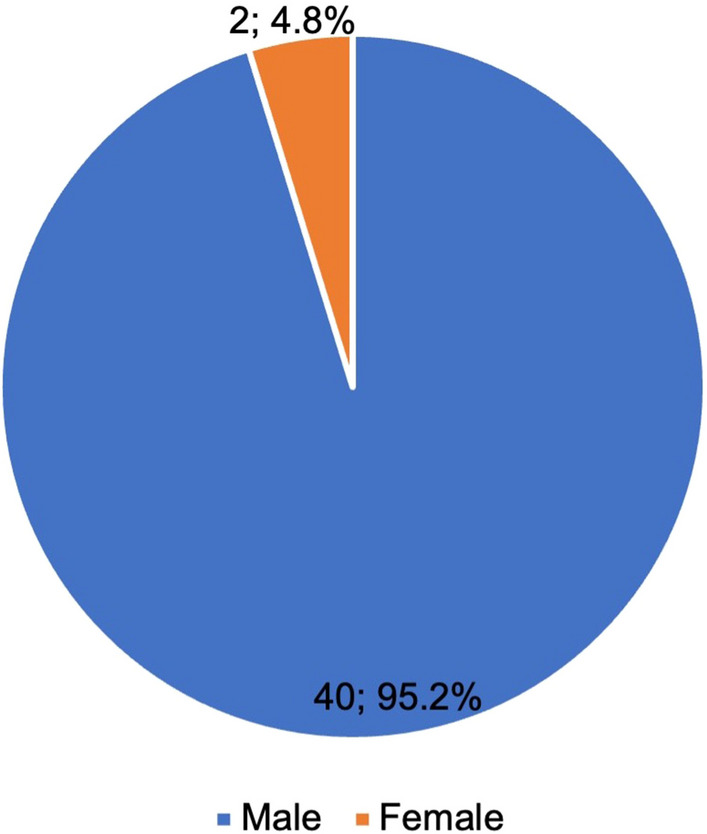
Gender distribution of the analyzed DGPT members. The sample sizes and percentages are given

Far more men than women made it into the analyzed data set (Fig. 4). Overall, there are 11.9% women in the data set of biologists and biochemists in Germany; in pharmacology, the figure is only 4.8% (see Table 2). This shows that the gender ratio in the analyzed data set is even more unequal.

**Fig. 5:**
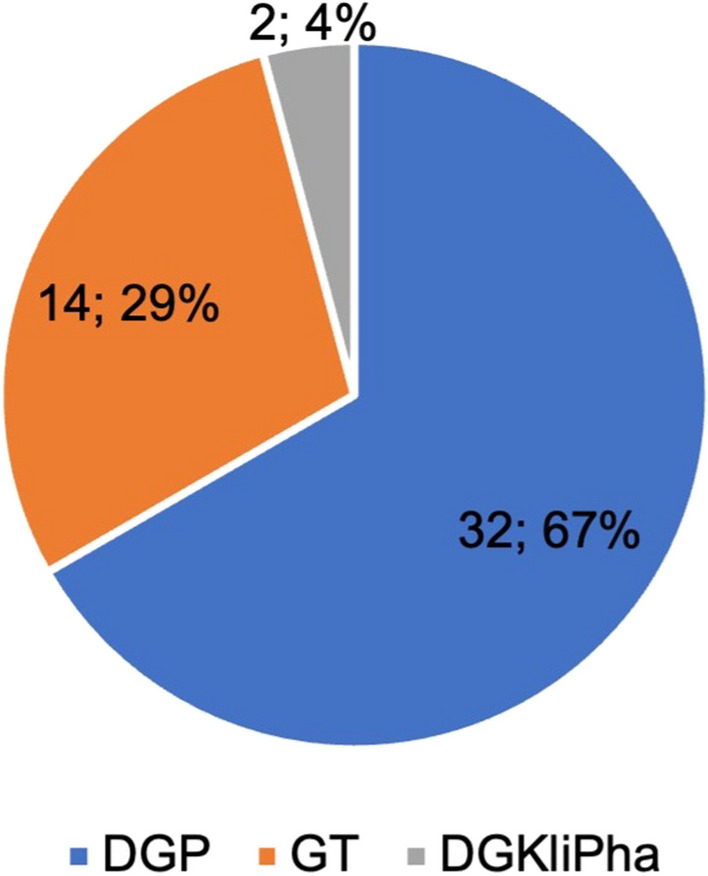
Distribution of scientists among the sub-societies of the DGPT. Please note that there are double memberships, so the number of memberships exceeds the total number of scientists. The sample sizes and percentages are given

Membership of the sub-societies was also recorded. As it is possible for an individual researcher to be a member of several sub-societies (Fig. 1), the total number of memberships in Fig. 5 exceeds the number of 42 analyzed persons. The double memberships in the analyzed cohort exist in DGP + GT and DGP + DGKliPha. Most of the researchers are members of the DGP, including the only two women on the list. The two memberships in the DGKliPha are not singular, but both are double memberships in the DGP. In reference to the total number of members in the sub-societies listed in Fig. 1, the relative proportion of elite members is calculated in Table 3. More DGP members have achieved elite status than members of the GT or DGKliPha. A total of 1.74% of DGPT members (see Fig. 1) have achieved the elite status defined in this analysis.

**Table 3:** Relative proportion of elite members of DGPT sub-societies to the total number of members. The membership numbers for the sub-societies include double and triple memberships, so the sum of the membership numbers of GT, DGP and DGKliPha exceeds the actual number of members of DGPT

(Sub-)society	Number of members	Number of elite scientists	Percentage of elite scientists
GT	1515	14	0.92%
DGP	658	32	4.86%
DGKliPha	310	2	0.65%
DGPT	2414	42	1.74%

Table 4 compares the findings of the present study with previous studies by Zehetbauer et al. (2022) and Zöllner and Seifert (2023). The previous studies examined the publication behaviour in the journals *Naunyn-Schmiedeberg’s Archives of Pharmacology* (peer-reviewed) and *Biospektrum* (non-peer-reviewed) over a certain period and, among other aspects, established year-specific, discipline-specific and geographical correlations. As the present analysis covers both historically and presently significant research and is less year-specific than the previous studies, the proportion of women is comparatively low. This should also be considered against the background of the data analyzed: due to vertical segregation, the proportion of women in elite research is once again considerably lower. The higher the status of the analyzed academics, the lower the proportion of women in the respective group.

**Table 4:** Comparison of the present study with the studies by Zehetbauer et al. (2022) and Zöllner and Seifert (2023)

Aspect	Zehetbauer et al. (2022)	Zöllner and Seifert (2023)	Present study
Data source	*Naunyn-Schmiedeberg’s Archives of Pharmacology*	*Biospektrum*	*Research.com*’s ‘best scientist’ ranking
Years analyzed	2000–2020	1999–2021	Data collection in 2022
Country	Germany	Germany	29 countries including Germany
Persons analyzed and results	2886 authors −2071 male (71.8%) −815 female (28.2%) Including 651 first authors −443 male (68.0%) −208 female (32.0%) And 651 senior authors −574 male (88.2%) −77 female (11.8%)	3197 authors −2147 male (67.2%) −1050 female (32.8%) Including 76 pharmacologists −62 male (81.6%) −14 female (18.4%)	9657 researchers in total −8217 male (85.1%) −1440 female (14.9%) Including 999 German researchers −880 male (88.1%) −119 female (11.9%) Including 42 members of the DGPT −40 male (95.2%) −2 female (4.8%)

### How a researcher gets included into the *Research.com* ranking: a matter of academic age

For a more precise age analysis, the age of the scientists in 2022 (year of data collection) was determined using the respective date of birth. The information was taken from CVs that were freely accessible on the internet. In cases where no dates of birth could be found, the DGPT’s membership administration kindly provided this information. To analyze the academic careers of the researchers in more detail, we defined the ‘academic age’. For this parameter, the age in 2022 was used and 25 years were subtracted to determine the ‘academic age’, because usually scientists do not publish before the age of 25 and their academic career does not begin until then. This determination made it possible to generate new bibliometric parameters, such as the quotient of the h-Index and ‘academic age’. This serves to compare the h-Index among researchers so that academic performance can be analyzed in terms of the length of the academic career. In Table 5, the main parameters of this age analysis are summarized. The mean h-Index/‘academic age’ quotient is 1.68. This means that the average (elite) DGPT member gains 1.68 h-Index points per year of academic career. Thus, approximately 24 years of active academic activity are necessary for a DGPT member to be included in the *Research.com* ranking (scientists with an h-Index of 40 or more are included in the list).

**Table 5:** Overview of the age analysis of the 42 DGPT members

Parameter	Value
Mean age (SD)	64.95 (9.84)
Min–max age	43–87
Mean ‘academic age’ (SD)	39.95 (9.84)
Min–max ‘academic age’	18–62
Mean h-Index/‘academic age’ (SD)	1.68 (0.81)

**Fig. 6:**
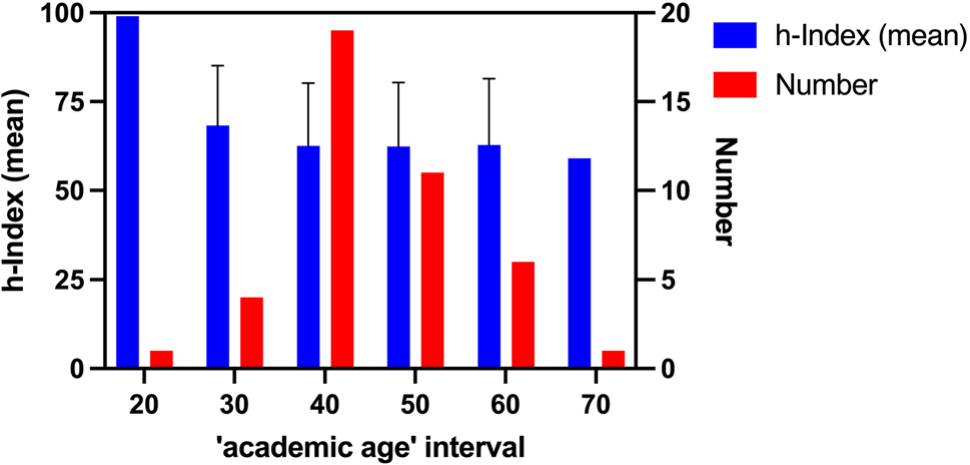
Age distribution of the 42 analyzed DGPT members. On the x-axis, the researchers are summarized in ‘academic age’ intervals (each up to the respective x-value). The blue bars reflect the mean h-Index of the interval on the left y-axis, the red bars the number of researchers in the interval on the right y-axis. Additionally, the error bars in black represent the standard deviation of the mean h-Index

**Fig. 7:**
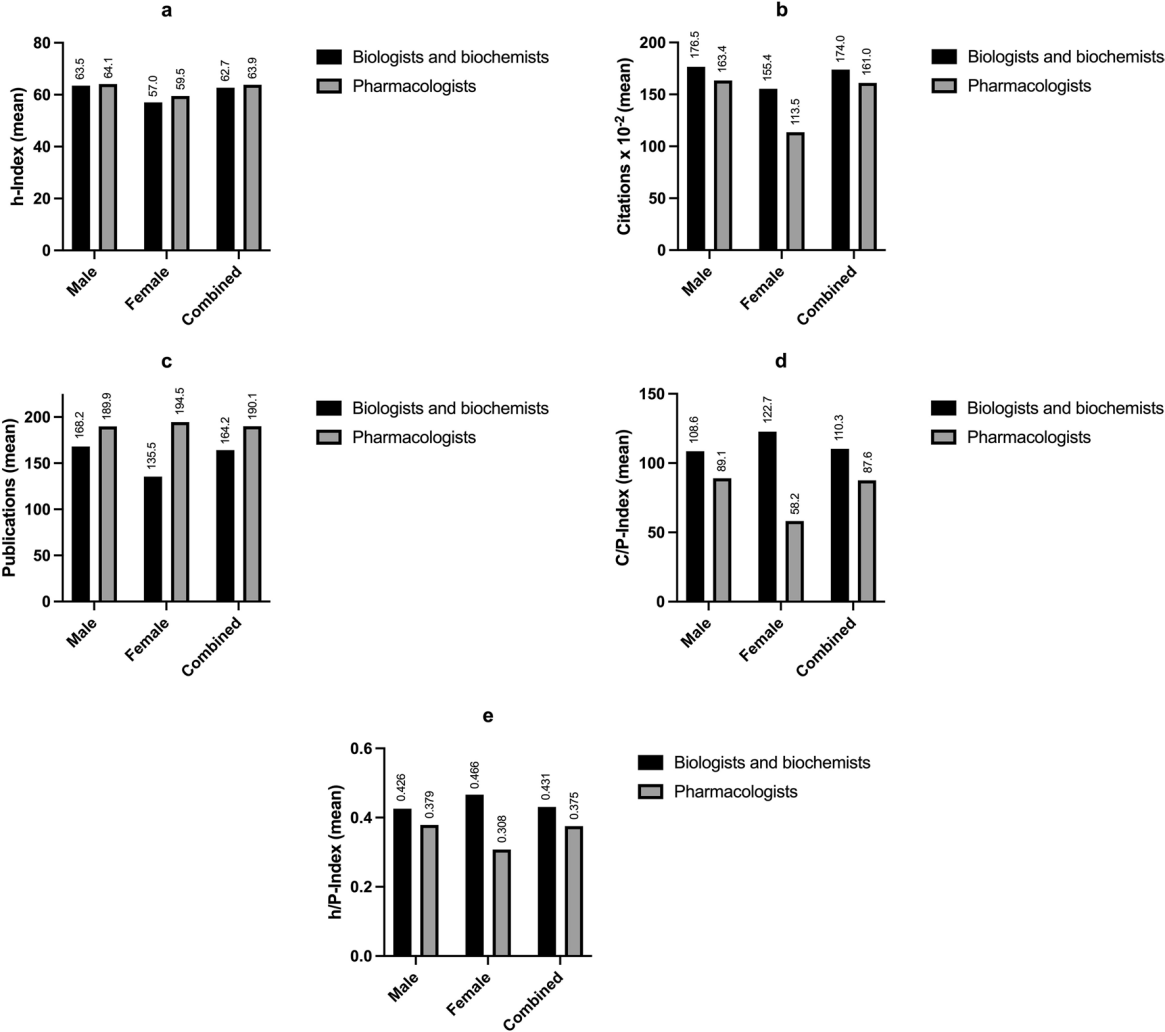
Comparison of the mean bibliometric parameters (**a** h-Index, **b** citations, **c** publications, **d** C/P-Index and **e** h/P-Index) between the German biologists/biochemists and the pharmacologists. Indicated at the top of the bars are the means of each group

Figure 6 shows the age distribution of the analyzed DGPT scientists. For this purpose, the scientists were grouped into academic age intervals on the x-axis according to the length of their academic career and the mean h-Index was then determined within these intervals. Most scientists are found in the age interval under 40 years and under 50 years, with the mean value for ‘academic age’ being 39.95. There are no large age-dependent differences in the h-Index, but it stabilizes at a level between 60 and 70 with a tendency to be slightly higher in the lower age intervals. This mean is also comparable with the mean value analysis in Fig. 7a. An exception to this is the scientist in the interval less than or equal to 20 academic years: as the interval here only consists of one person, however, the significantly larger h-Index is less meaningful. These results support the statement that the h-Index tends to be slightly higher among younger DGPT members and that there is another small peak at around 50 years of academic career duration.

### Comparison of DGPT members with Nobel laureates

A previous study by Bünemann and Seifert (2024) dealt with a bibliometric analysis of Nobel laureates from 2006 to 2022 in physiology or medicine and chemistry with a pharmacological relevance. Although this analysis covers a different ‘target group’, i.e., that of the Nobel laureates, the data collected are suitable for comparing the two cohorts with each other. Table 6 summarizes these results. The results of the present study are not listed separately for males and females, as the female cohort consists of only two persons. The results of the DGPT members are therefore summarized. The ages of the scientists analyzed are roughly comparable. On average, the Nobel laureates are characterized by a higher h-Index (also age-adjusted as a quotient of h-Index and age) and have more publications than the elite DGPT scientists. Among the latter group, there is no Nobel laureate. Thus, regarding bibliometric parameters, Nobel laureates as a group can be clearly distinguished from elite DGPT members. Evidently, Nobel laureates are the most prominent group of researchers globally. Thus, it is not surprising that their h-Indices are higher than those of the top DGPT members. But as will be discussed below, discipline-specific aspects may play a role because most Nobel laureates analyzed by Bünemann and Seifert (2024) are not pharmacologists but rather biochemists and chemists.

**Table 6:** Comparison of the present study with the study by Bünemann and Seifert (2024)

Parameter	Bünemann and Seifert (2024) (Nobel laureates)		Present study (elite DGPT members)
	Female	Male	
Mean age	60.1	67.4	64.95
Mean h-Index	78.78	89.79	63.86
Mean age-adjusted h-Index (SD)	1.311 (0.418)	1.238 (0.250)	0.999 (0.275)
Mean publications	321.5	333.3	190.1

### Bibliometric differences between pharmacology and biochemistry

**Fig. 8:**
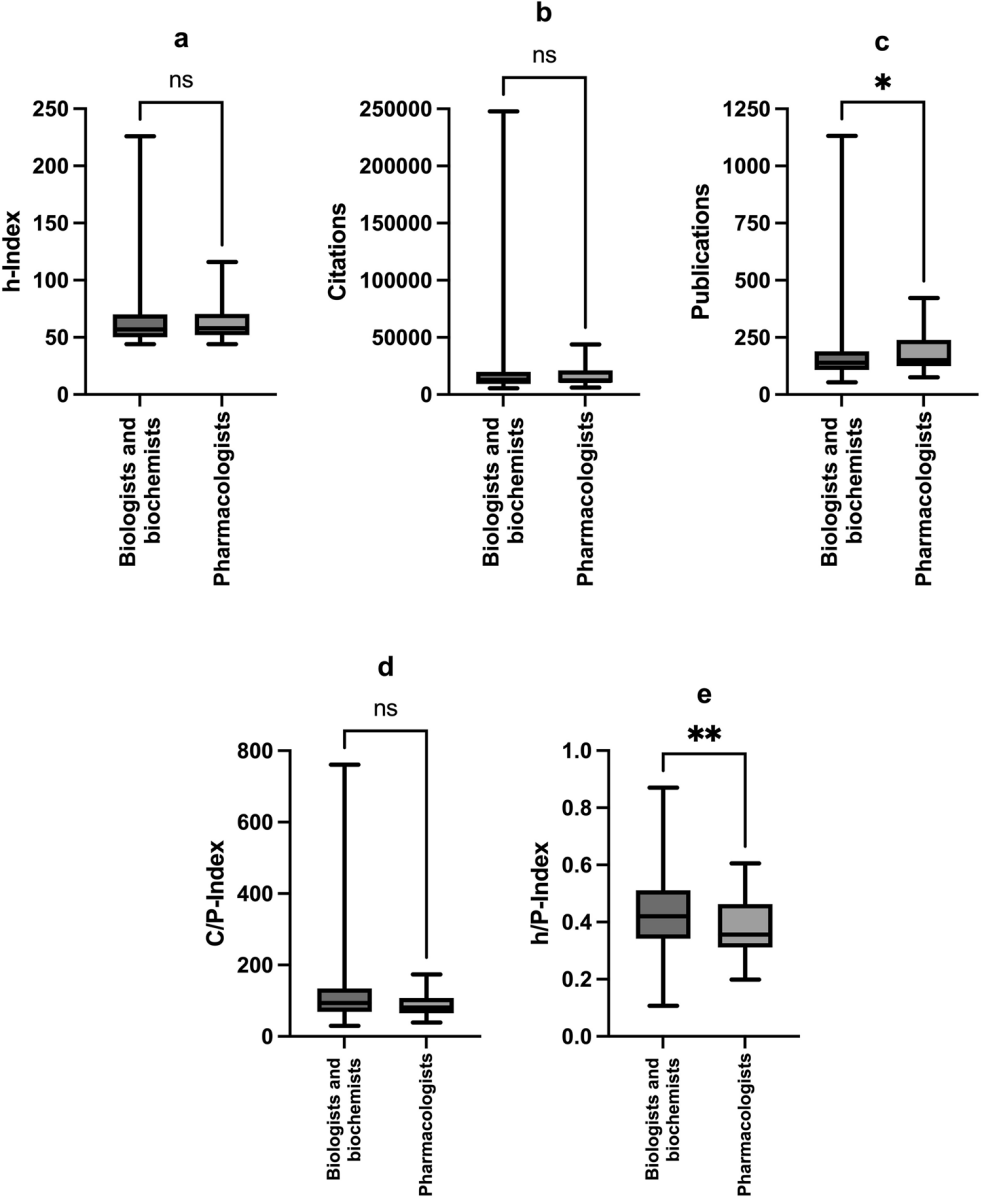
h-Index (**a**), citations (**b**), publications (**c**), C/P-Index (**d**) and h/P-Index analysis (**e**) of the German biologists and biochemists vs. pharmacologists. Legend: ns = not significant (*p* > 0.05); *p ≤ 0.05; **p ≤ 0.01. The whiskers represent the range from minimum to maximum value, the gray boxes extend from the 25th to 75th percentiles and the black horizontal line is the median

Compared to biochemistry and biology, pharmacology is a rather small field (see Table 2; Fig. 3). Therefore, the question arises whether a small scientific discipline may result in ‘poorer performance’ in bibliometric analyses than a larger field. A smaller scientific field may offer fewer opportunities for the individual researcher, but on the other hand, also less competition. A less popular field does not receive as much attention in the scientific community as a more popular field. This tends to lead to fewer citations that researchers receive for their publications. These connections are illustrated in Fig. 7a–e. The bar graphs summarize the mean values of the evaluated bibliometric parameters for the group of pharmacologists vs. the other biologists and biochemists. Figure 7a shows that the mean values of the h-Index do not differ greatly: The picture is relatively balanced—both between biologists/biochemists and pharmacologists and to a certain extent between the genders. Figure 7b shows that pharmacologists have fewer total citations on average, but more total publications (Fig. 7c). This results in a lower C/P-Index for the pharmacologists (Fig. 7d), as well as a lower h/P-Index (Fig. 7e). The differences in the bibliometric parameters for the combined groups (not for the individual genders because of the small sample size of female pharmacologists) were tested for significance. The non-parametric Mann-Whitney test was carried out in each case, as the data did not show a normal distribution. Significant differences can be found in the publications and the h/P-Index. Pharmacologists publish significantly more than biologists and biochemists, which also explains the significantly lower h/P-Index for pharmacologists in comparison to biologists and biochemists. These relations are shown in Fig. 8a–e and summarized in Table 7.

**Table 7:** Statistical analysis of the bibliometric parameters for German biologists and biochemists vs. pharmacologists

Bibliometric parameter	Mean biologists and biochemists (SD)	Mean pharmacologists (SD)	*p *value
h-Index	62.7 (19.0)	63.9 (17.5)	0.4413
Citations	17,397 (15,318)	16,105 (9135)	0.7131
Publications	164.2 (94.0)	190.1 (93.9)	0.0407 (*)
C/P-Index	110.3 (64.3)	87.6 (28.7)	0.0529
h/P-Index	0.431 (0.126)	0.376 (0.101)	0.0053 (**)

*p ≤ 0.05; **p ≤ 0.01

### Are pharmacologists more productive than toxicologists?

**Fig. 9:**
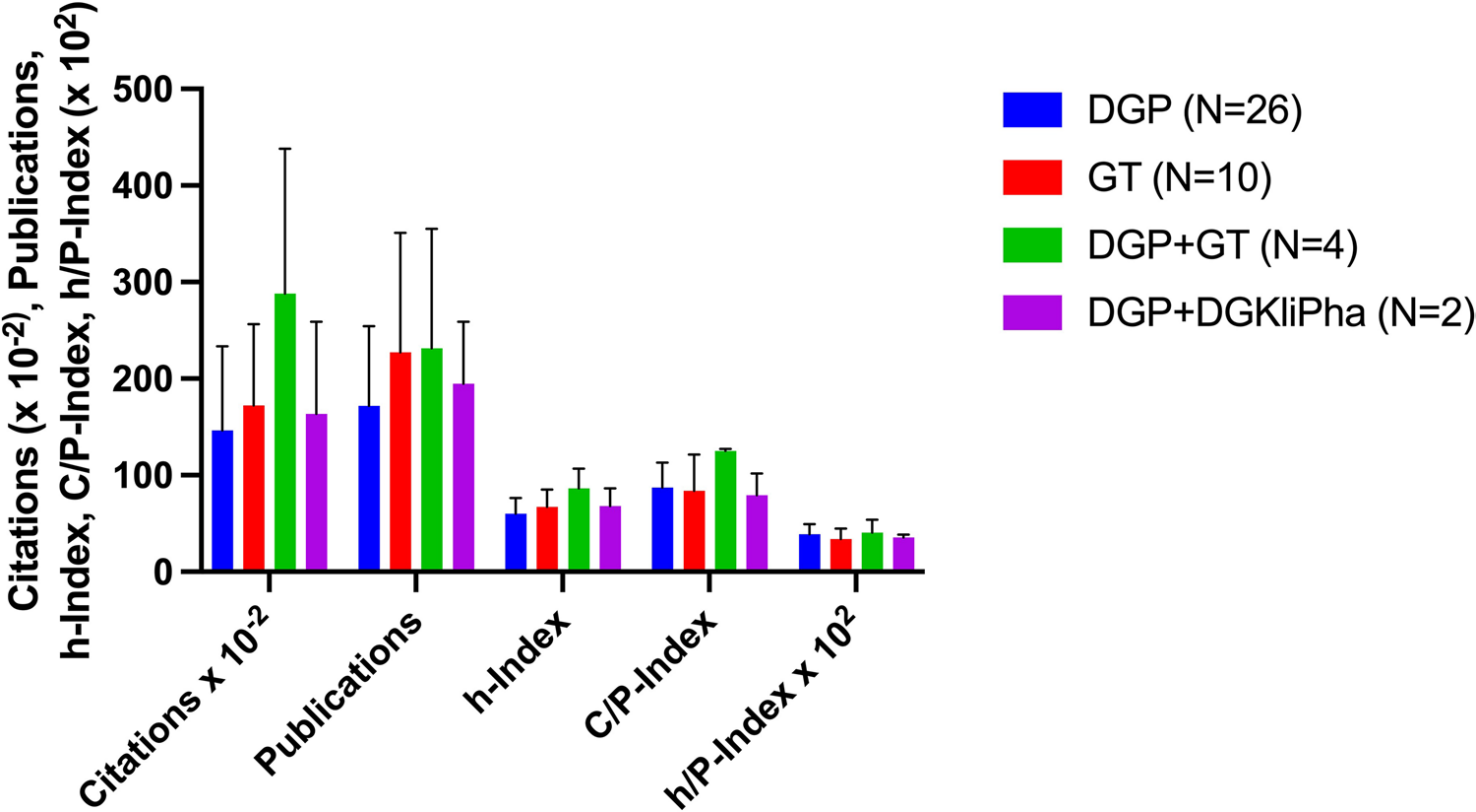
Overview of the means of the bibliometric parameters of the sub-societies of the DGPT. The error bars in black represent the standard deviation

There are some double memberships for the 42 analyzed elite scientists of the DGPT: these exist four times in a double membership in the German Society for Pharmacology and the Society for Toxicology, and two scientists are members of both the German Society for Pharmacology and the German Society for Clinical Pharmacology. The main comparison addresses individual memberships in the DGP and the GT. Figure 9 shows the mean values of the analyzed bibliometric parameters for the sub-societies. The statistical analysis, which was carried out more precisely only for the members of the DGP and the GT due to the small sample size for DGP + GT and DGP + DGKliPha, shows no statistical significance. For statistical analysis, the citations, publications, the h-Index and the C/P-Index were analyzed using a Mann-Whitney test due to the non-parametric distribution, and the normally distributed h/P-Index was analyzed using an unpaired *t*-test. The results are summarized in Table 8. This clearly shows that elite toxicologists are similarly productive as elite pharmacologists.

**Table 8:** Overview of the statistical analysis of the bibliometric parameters of the two bigger sub-societies DGP and GT (DGP 26 scientists, GT 10 scientists)

Bibliometric parameter	Mean DGP (SD)	Mean GT (SD)	*p *value
Citations × 10^−2^	146.6 (87.0)	172.3 (84.4)	0.3197
Publications	171.9 (82.4)	227.3 (123.9)	0.1915
h-Index	60.2 (16.3)	67.1 (18.2)	0.2371
C/P-Index	87.4 (25.7)	84.2 (37.1)	0.4330
h/P-Index × 10^2^	38.9 (10.5)	34.0 (10.7)	0.2213

The number of elite toxicologists is substantially smaller than the number of elite pharmacologists (Tables 3 and 8) although the GT has considerably more members than the DGP. This apparent discrepancy is explained by two factors. Firstly, the number of toxicology professorships in Germany is considerably smaller than the number of pharmacology professorships, reducing the pool of potential academically active toxicologists. Secondly, a major mission of the GT is to provide toxicological services to the chemical and pharmaceutical industry in Germany (see also Introduction), a field that provides fewer opportunities for scientific work than academia. One can only compare the academically active toxicologists and pharmacologists, and here, there are no differences among the groups. The similarities of the bibliometric data show that pharmacology and toxicology are closely related scientific fields with a similar culture, belonging together.

### The Günter Schultz school *versus *the Franz Hofmann school

German pharmacology can be categorized into various ‘schools’, two major ones being those of Günter Schultz (https://de.wikipedia.org/wiki/Günter_Schultz) and of Franz Hofmann (https://de.wikipedia.org/wiki/Franz_Hofmann_(Mediziner). The term ‘pharmacological schools’ refers to highly influential and scientifically productive pharmacology professors in Germany bringing numerous of their postdocs and/or doctoral students into top academic positions.

Günter Schultz (1936–2021) was a highly influential German pharmacologist who had a major impact on the discipline in Germany. He was initially a professor in Heidelberg (1973–1983) until he took over the chair at the Freie Universität Berlin in 1983 until his retirement in 2003. Franz Hofmann (born in 1942) also spent a long time researching in Heidelberg (1970–1985) before moving to the University of the Saarland (1985–1990) and then to the Technical University of Munich (1990–2008). Numerous pharmacologists obtained their habilitation under Schultz and Hofmann and later became pharmacology professors themselves. A large part of current German pharmacology was therefore shaped by the schools of Günter Schultz and Franz Hofmann. Further very detailed information can be found in the works of Athineos Philippu on the pharmacological institutes in Germany (Philippu 2004) and on the excellent *Wikipedia* accounts on both researchers. A total of 7 scientists in the *Research.com* data set can be assigned to the Günter Schultz school and 6 scientists to the Franz Hofmann school (including Schultz and Hofmann for the respective group).

**Fig. 10:**
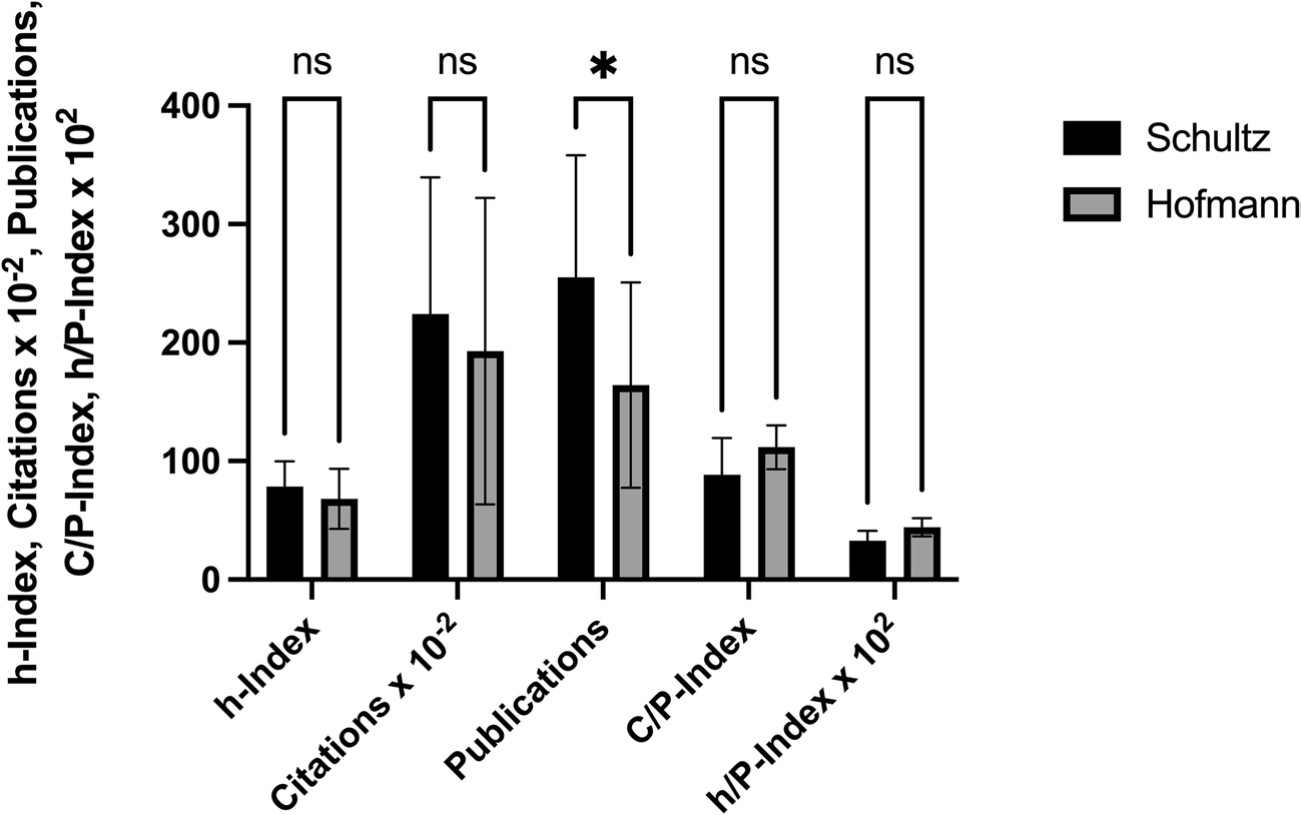
Statistical comparison of two big German pharmacological schools (Schultz vs. Hofmann). Legend: ns = not significant (*p* > 0.05); *p ≤ 0.05. The error bars in black represent the standard deviation

The comparability of the two schools is given, as the sample sizes in the present data set are similar, the members are mainly in the DGP and the age range is similar. A two-way ANOVA was carried out for the statistical analysis. The results of the analysis are shown graphically in Fig. 10 and summarized in Table 9. Significance can be seen: the Schultz school publishes significantly more than the Hofmann school (*p* = 0.0250). The remaining correlations are not significant.

**Table 9:** Overview of the means of the bibliometric parameters of the two big pharmacological schools (Schultz 7 scientists, Hofmann 6 scientists)

Bibliometric parameter	Mean Günter Schultz school (SD)	Mean Franz Hofmann school (SD)	*p *value
h-Index	78.6 (21.2)	68.2 (25.4)	0.7935
Citations × 10^−2^	224.1 (115.3)	192.8 (12.9)	0.4312
Publications	255.3 (102.7)	164.2 (86.8)	0.0250 (*)
C/P-Index	88.6 (30.9)	111.7 (18.6)	0.5627
h/P-Index × 10^2^	32.9 (8.12)	44.1 (7.60)	0.9978

*p ≤ 0.05

### The popular *Laborjournal *ranking

In a further evaluation approach, the scientist ranking on the website www.laborjournal.de was checked for matches with the present data set from this analysis (Rembold 2017; Rembold 2021). The *Laborjournal* rankings are highly popular in Germany. In each issue, a separate scientific field is analyzed by the *Laborjournal*, and the rankings are then used (evidently by the most highly ranked scientists) to promote their rank in the respective field, e.g. by publications on the rank in local university journals or by posting the rank in online CVs. Thus, informally, over the years, *Laborjournal* has obtained high authority on scientific rankings in Germany. *Laborjournal* has a wide reach in the German scientific community since it is distributed free-of-charge in research institutes and can also be downloaded free-of-charge. The costs for *Laborjournal* production are covered by placement of advertisements for research-related products and research positions.

The pharmacology ranking of *Laborjournal* is from 2017 and lists the most cited researchers in pharmacology between 2011 and 2015 (50 researchers listed in total) (Rembold 2017). In a separate ranking from 2021, the most cited researchers in toxicology between 2010 and 2019 (30 researchers listed in total) were analyzed (Rembold 2021). Most surprisingly, there were only 2 matches per list with the *Research.com* (2022) data set so that only 4 of the top active members of the DGPT can be found in the ranking of *Laborjournal*. Due to the extremely low match between both rankings, we did not further compare *Laborjournal* and *Research.com* rankings. This once again highlights the problem of bibliometric analyses in two dimensions: firstly, the importance of defining a discipline and clearly assigning academics to corresponding disciplines for the purposes of analysis and comparability and, secondly, the difficulty of using the ‘right’ or ‘best’ parameter for the analysis.

### Individual publications patterns: publications per year (PPY)

**Fig. 11:**
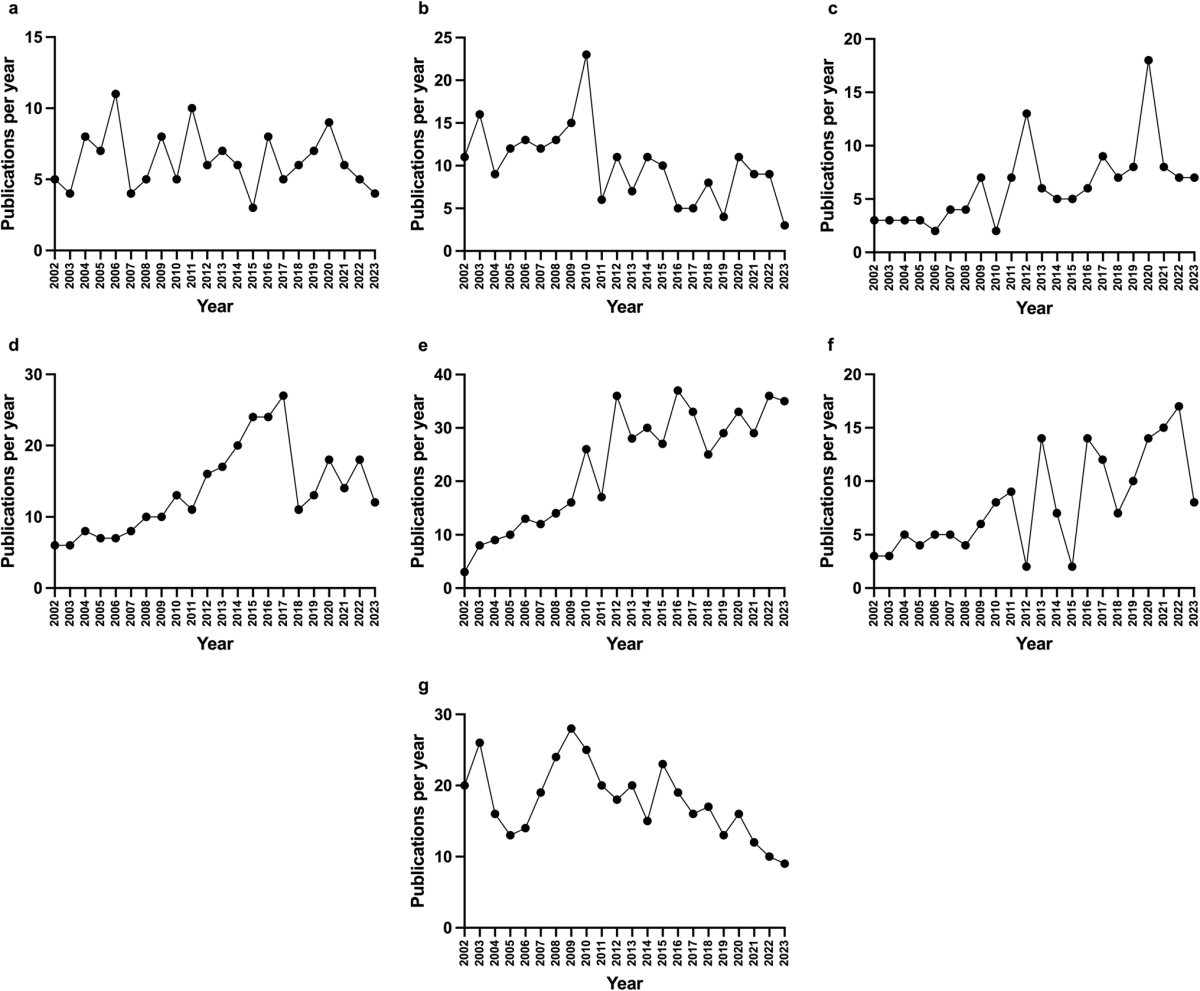
Illustration of the pattern of publications per year of selected researchers between the years 2002 and 2023 (years 2002–2023 on the x-axis, publications per year on the y-axis). Various patterns can be recognized: fluctuating fairly evenly around a mean value (**a**); one (**b**) or several peaks present (**c**); rising to a peak, then falling (**d**); rising and remaining relatively constant (**e**); rising inconstantly (**f**) and falling (**g**)

To illustrate the publication behavior of the examined scientists, an analysis of publications per year (PPY) in the period 2002–2023 was carried out using *PubMed* data via an author search. This period was chosen because the authors were assigned with their full first names from 2002 onwards; as the year 2024 has not yet ended, the period up to 2023 was selected. The analysis took place in the age segment between 55 and 70 years (in 2024; 28 researchers in total), as this age cohort (‘pharmacological/toxicological baby boomers’) is strongly represented and can be easily divided into further subcategories (see below). After the corresponding search query in *PubMed* (‘last name, first name [Author]’), the visualization of the PPY was downloaded as a file and imported into Excel so that a graph of the chronological course of the PPY could be created for each scientist. The individual progressions can be categorized into patterns that are recurring. Seven patterns were defined in this study (see Fig. 11a–g; Table 10). Figure 11a shows an example of a scientist whose publications fluctuate with an evenly distributed mean value. The analysis in Table 10 shows that this tends to characterize researchers who have fewer publications overall. Figure 11b shows the pattern of a scientist with a clear peak; otherwise, the publications fluctuate relatively evenly; Fig. 11c shows a similar pattern with two or more distinct peaks. The pattern in Fig. 11d—i.e. increasing and then decreasing after reaching a peak—is increasingly evident in researchers who publish a lot. The pattern in Fig. 11e, increasing and then remaining constant at a high level, was only observed once. The trend towards an increase in PPY with some fluctuations over the years is shown in Fig. 11f. The tendency for PPY to decrease, as in Fig. 11g, is increasingly evident in researchers who belong to the older cohort—publications decrease towards the end of their career. In a further approach, the biannual publication rate, i.e. publications per 2 years (PP2Y), was recorded. Here, the seven patterns described are found again, the allocation remains largely the same, but there are individual scientists who change categories in the biannual analysis. In the larger interval, individual outliers are not as pronounced, so the patterns can change slightly. However, the basic patterns are the same.

**Table 10:** Overview of analyzed parameters in the cohort grouped in the patterns of Fig. 11a–g

Pattern as in Fig. 11	Mean age (SD)	Mean publications (SD)	Number of scientists (PPY)	Number of scientists (PP2Y)
a: fluctuating fairly evenly around a mean value	62.67 (3.67)	134.00 (34.71)	6	5
b: one peak	64.25 (6.40)	178.00 (95.29)	4	3
c: several peaks	58.80 (3.11)	155.80 (82.08)	5	3
d: rising to a peak, then falling	61.80 (2.05)	244.20 (61.71)	5	4
e: rising and remaining relatively constant	59.00	341.00	1	3
f: rising inconstantly	60.00 (1.00)	175.67 (56.00)	3	4
g: falling	65.25 (6.85)	179.75 (134.59)	4	4

Note that mean age and publications with SD are given for the grouping by PPY

**Fig. 12:**
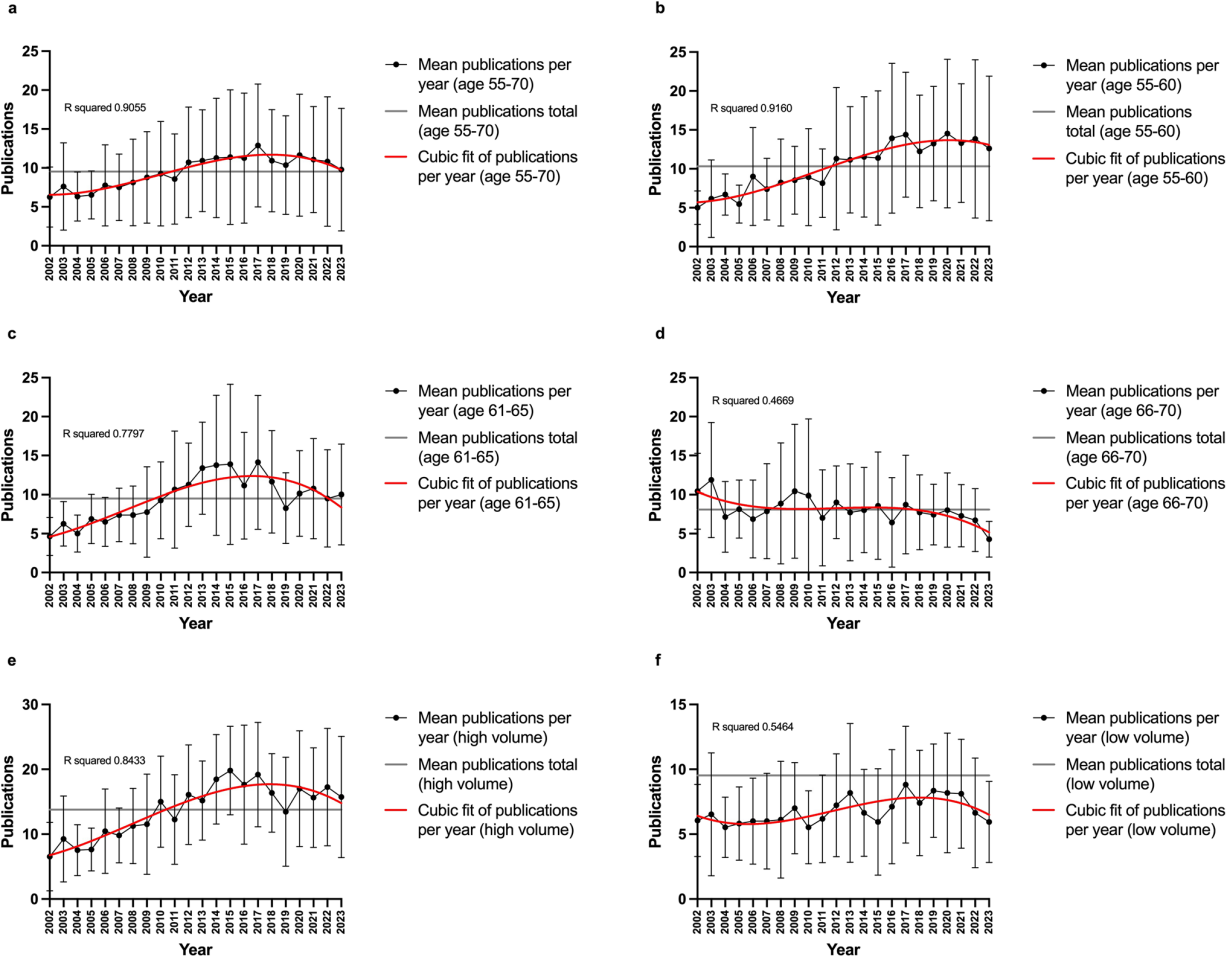
Summarized trends of average publications per year (years 2002–2023 on the x-axis, publications on the y-axis) in different analyzed groups: age group 55–70 (**a**); age group 55–60 (**b**); age group 61–65 (**c**); age group 66–70 (**d**); high volume publisher (**e**); low volume publisher (**f**). In black the connected data points, in grey the mean value of the respective group across all years, in red the cubic fit of the data points. Additionally, the error bars in black represent the standard deviation

Figure 12a–f shows summarized PPY progressions in different groups. Table 11 shows the statistical results of the analyses. The values for the PPY in the individual years were used to form a mean value, which was then used as the *y*-value. Figure 12a summarizes all 28 researchers analyzed in the 55–70 age cohort. There is an increase in productivity after 2002, which reaches a peak in 2017 and then declines again—in other words, it behaves according to the pattern in Fig. 11d. A more detailed sub-analysis in smaller age groups provides a more differentiated picture. Firstly, the group between 55 and 60 years was analyzed in Fig. 12b—the scientists here are fully active and still several years away from retirement. Productivity measured in terms of PPY increases on average over the years and is also higher than in Fig. 12a; it is also rather like Fig. 11e, i.e. a plateau in the recent years. Productivity will presumably decline again in the further course of the career. This can be seen in the cohort aged 61–65 in Fig. 12c: productivity rises to a peak around 5 years before that of the younger cohort. After that, a clear drop is recognizable. The curve thus corresponds to the one shown in Fig. 11d. The oldest cohort, i.e. the age between 66 and 70 years (official retirement), is shown in Fig. 12d and is characterized by decreasing PPY (like Fig. 11g). In retirement, publications decline, so the trend is downward. Over the years, the mean value of the PPY also decreases (decreasing from Fig. 12b–d). It can be estimated from these numbers that elite German pharmacologists and toxicologists reach their publication peak at an age of around 55 years before publication productivity declines. However, the large SD values in Fig. 12 illustrate that there are very large interindividual differences in publication productivity.

**Table 11:** Overview of the statistical analysis of the scientists’ number of publications per year in different analysis groups

Group (as in Fig. 12)	Mean PPY	SD	Significance (*p *value)
Age 55–70 (a)	9.54	6.69	/
Age 55–60 (b)	10.32	7.36	b vs. c: no (> 0.9999) b vs. d: yes (0.0070)** c vs. d: no (0.1023)
Age 61–65 (c)	9.52	6.20	
Age 66–70 (d)	8.10	5.65	
High-volume publisher (e)	13.78	7.83	e vs. f: yes (< 0.0001)****
Low-volume publisher (f)	6.79	3.88	

**p ≤ 0.01; ****p ≤ 0.0001

### High-volume and low-volume publishers

For Fig. 12e, f, the researchers were divided into two groups: high-volume publishers and low-volume publishers. For this purpose, the mean value of the total publications in the analyzed cohort of the 28 scientists was first determined (182.25), and then, the groups were categorized (larger or smaller than the mean). The high-volume publishers in Fig. 12e show a strongly increasing trend with a high average value of PPY, whereas the low-volume publishers (Fig. 12f) show less pronounced differences and smaller fluctuations. The mean value of the PPY calculated over all years is roughly halved for the low-volume publishers.

**Fig. 13:**
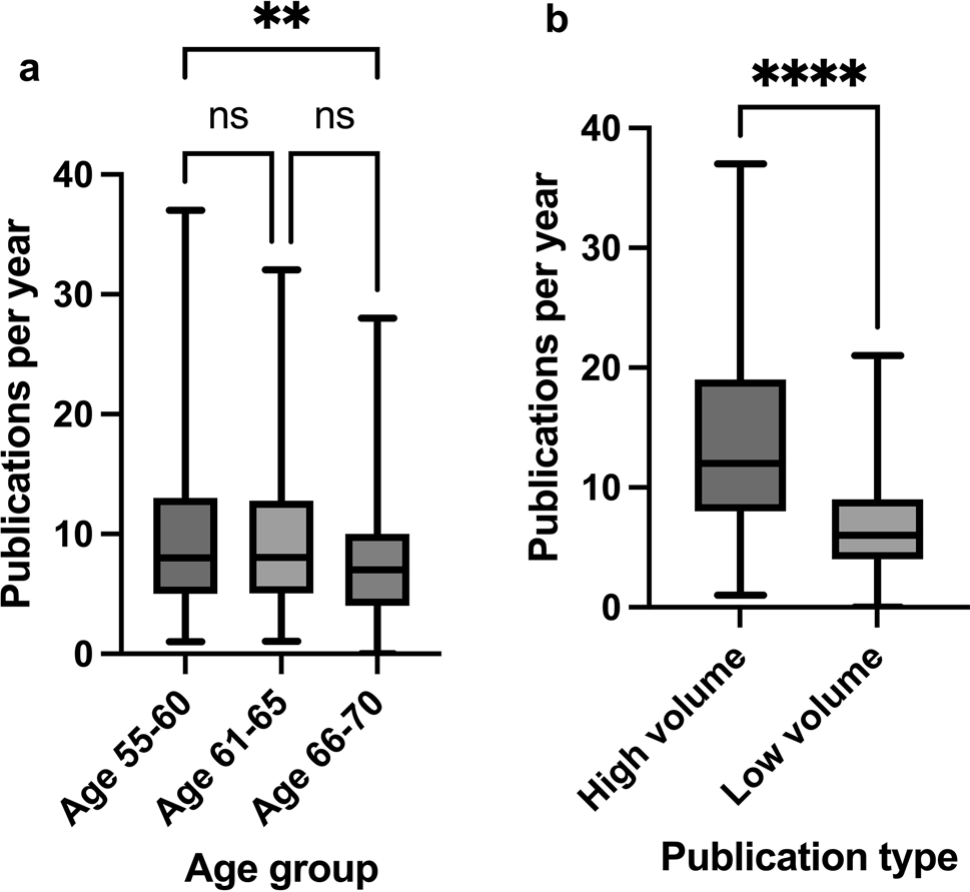
Statistical analysis of publications per year in the different age groups (**a**) and publication types (**b**). Legend: ns = not significant (*p* > 0.05); **p ≤ 0.01; ****p ≤ 0.0001. The whiskers represent the range from minimum to maximum value, the gray boxes extend from the 25th to 75th percentiles and the black horizontal line is the median

A detailed statistical analysis of the different age cohorts and publication types is carried out in Fig. 13a, b; the results are listed in Table 11. A non-parametric test, the Kruskal-Wallis test, was carried out for the age analysis due to the lack of a normal distribution. There is a decrease in the mean PPY over the years, but the difference is only significant between the 55–60-year-old researchers and the 66–70-year-old researchers (*p* = 0.0070). The other correlations are not significant. The Mann-Whitney test was applied to the non-normally distributed data between the high- and low-volume publishers. The difference in the mean value of the PPY is highly significant (*p* < 0.0001) and higher for the high-volume publishers.

### Individual factors contributing to publication activity

The analysis of the PYY and publication volume is intended to demonstrate how individually academic careers proceed. There can be numerous reasons for interindividual differences in PPY, for example family issues, health problems or professional reasons such as shifted main activities (extensive teaching obligations, writing or editing textbooks or handbooks, taking over academic administrative positions and the university such as dean or at DGPT or Deutsche Forschungsgemeinschaft (DFG) level, journal editor commitments) or the size of the working group (successful or unsuccessful grant applications) and the presence (or absence) of talented junior scientists in the working group who can write good first paper drafts. The popularity of a research topic may also impact publication and citation records. Scientists may decide not to ‘waste time’ to publish ‘negative’, ‘descriptive’ or ‘confirmatory’ data. Other scientists may elect not to ‘spoil their average impact factor’ by publishing in lower impact factor journals. Typically, moving from one academic position to another academic position in a different city is associated with a temporary decline in academic productivity as laboratory operations are disrupted. Personal publication preferences play a part as well. Some researchers prefer long and comprehensive papers, whereas as other researchers prefer to publish smaller advances. Complex interdisciplinary projects involving experiments with gene knockout animals or crystallography take more time to complete than cell culture experiments or studies using fewer methodologies. For publications in top-impact factor journals, often, months if not years of work may have to be invested into a revision until the paper becomes accepted. These important factors affecting publication productivity are captured only to a small extent in bibliometric analyses.

Against the background of bibliometric analyses, a further aspect becomes apparent that is lost in the generalization of academic performance: changes in academic productivity occur, particularly depending on age, as explained above. For example, PYY decreases with advancing age, while at the beginning of a career a researcher will presumably have fewer total publications, citations and thus also a lower h-Index than at a later point in their career. Factors such as the age-specific h-Index as a quotient of h-Index/‘academic age’ should take such contexts into account.

### Is the *Research.com *ranking suitable for identifying ‘the best’ pharmacologist?

**Fig. 14:**
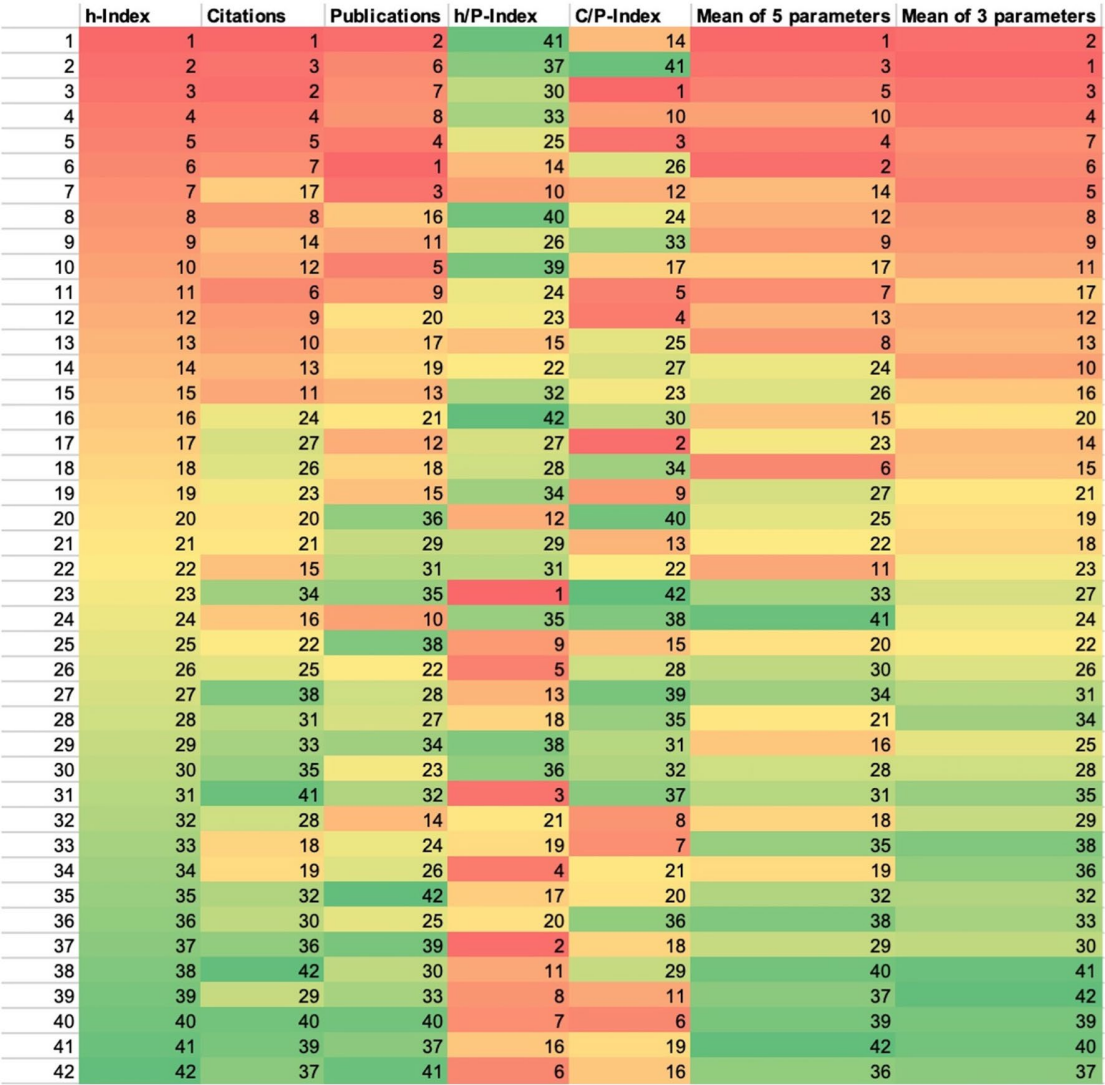
Ranking of the 42 DGPT members according to the various bibliometric parameters to illustrate the lack of comparability between different bibliometric analyses. Researchers are listed from 1–42 according to their h-Index. Each researcher was assigned an h-Index color, ranging from dark red over yellow to dark green. The scientists were then ranked to other bibliometric parameters, keeping their original h-Index color. The mean of 5 parameters refers to equally weighted h-Index, citations, publications, h/P-Index and C/P-Index. The mean of 3 parameters refers to equally weighted h-Index, citations and publications

Figure 14 illustrates the inherent problems of the bibliometric analyses. The 42 analyzed DGPT members were sorted according to the various analysis parameters. Firstly, they were ranked from 1 to 42 according to the h-Index, as is the case in the ‘best scientist’ ranking (year 2022) on *Research.com*. According to this ranking, the researchers were assigned a number and a color using conditional formatting in Excel. This creates a traffic light scheme from red (high position in the h-Index ranking) to yellow to green (low position in the h-Index ranking). This color and the number are permanently assigned to the corresponding scientist for the following rankings. Overall, the researchers are sorted according to all the bibliometric parameters surveyed: h-Index, citations, publications, h/P-Index, C/P-Index (from left to right in Fig. 14). Finally, calculations were made to determine the average ranking of a researcher taking into account the 5 parameters (h-Index, citations, publications, C/P-Index, h/P-Index) or 3 parameters (h-Index, citations, publications) and the ranking was repeated on the basis of these calculations. The result is a colorful and mixed picture: While the color sequence for the citations and publications is still roughly the same, the picture for the new ‘secondary parameters’—i.e. the h/P-Index and the C/P-Index—is clearly different. In the case of the h/P-Index, it even seems to be in the opposite order. This shows that the different analysis parameters illuminate different aspects. Specifically, we have four candidates for position 1 and six candidates each for positions 2 and 3 in the ranking. Scientist 1 is listed 5 times in the various rankings, while scientists 2 and 3 are listed four times each. But with certain bibliometric parameters, even ‘bottom-ranked’ scientists such as 30, 37 and 41 appear in the top 3. Thus, ranking of scientists in our case study arbitrary.

The ranking according to the mean values was carried out to show that consideration of all parameters creates a more comprehensive and balanced picture. For fair analyses and comparisons between scientists, all aspects of a person’s scientific work must be considered. Kaur et al. (2013) describe this connection, which is also shown in Table 1. Ideally, normalized metrics are necessary for better comparability. The traffic light illustration of the mean values also shows that the differentiation between ‘top’ and ‘bottom’ scientists works well with a comprehensive view of the primary bibliometric parameters. However, the very broad mid-range of the analyzed scientists is rather arbitrary.

Modern science is based on the evaluation of research (Bornmann 2011). This can take place in various ways. Firstly, the quality and significance of scientific content and research results are selected via the assessment of peer-reviewed journals. Bibliometrics are used for the factor-based assessment of science, which has become an integral part of science. Various evaluation parameters exist for this purpose, each of which has advantages and disadvantages. The basis for many parameters is the total citations, which according to Bornmann and Marx (2014) have a high level of precision as they are based on the judgement of many scientists. They compare it to the wisdom of crowds. However, what is not reflected in the total citations is the significance and usefulness of the scientific result (Bornmann and Marx 2014). The other analysis parameters must therefore not only be considered against the background of their own disadvantages, but the citations must also be kept in mind.

The h-Index favours ‘those authors who produce a series of influential papers rather than those authors who either produce many papers that are soon forgotten or produce a few that are uncharacteristically influential’ (Kelly and Jennions 2006). But it should also be kept in mind that numerous papers, referred to as ‘sleeping beauties’ (van Raan and Winnink 2019), are not recognized in their values many years (or even decades) after publication. Sleeping beauties may be captured only in conventional bibliometric analyses of researchers with high academic age, potentially only after retirement or even long after death.

### Limitations

Pharmacology is a cross-sectional science and has relations to numerous scientific fields including biochemistry, biophysics, molecular biology, cell biology, genetics, immunology, neurosciences, informatics and clinical fields such as cardiology, oncology and psychiatry. Thus, depending on the focus of research, some pharmacologists may be listed in different categories than in the *Research.com* category on biology and biochemistry. However, we did not want to manipulate the raw data set from *Research.com*. This study is not meant to feature the achievements of individual pharmacologists; it is rather meant as a case study regarding the advantages and disadvantages of bibliometric analyses.

Another limitation of our study concerns the mandatory membership in the DGPT to be included in this analysis. Evidently, membership in the DGPT signals close association to the discipline, but the terms ‘pharmacology’ and ‘toxicology’ are used rather broadly in the general scientific discussion. Some researchers may consider themselves as pharmacologists and toxicologists (because they work with drugs, toxins, or poisons), although they are not DGPT members. These researchers were, however, not included in our analysis.

Thus, based on the above two limitations, we analyzed only a clearly defined ‘hard core’ of German pharmacology and toxicology but not its broad intersecting areas with other disciplines.

### Conclusions

Women are clearly underrepresented among top pharmacologists, which can probably also be attributed to vertical segregation. The low proportion of women is much more noticeable among pharmacologists than among biologists and biochemists. Pharmacologists have a different international perception than biologists and biochemists, which is reflected in a significantly lower h/P-Index. Within two big German ‘pharmacological schools’, there are no major differences in scientific output, the only significance is in the number of total publications between the Schultz and Hofmann schools. Elite pharmacologists and elite toxicologists are similarly productive in terms of bibliometric parameters. Thus, from the bibliometric analysis, these two fields of research belong together.

Our study highlights that there is no ‘perfect’ bibliometric parameter for ranking of scientists. It depends on the specific point of view, and with some parameters, ‘bottom’-ranked scientists may pop at in the absolute top. Particularly in the large broad middle segment of elite researchers, bibliometric parameters are very arbitrary. Thus, we hope that our study is helping research institutes and funding organizations making wise decisions about hiring the right person and making the best funding decision, respectively. Just looking on a single bibliometric parameter is certainly not the best advice for making wise decisions. Looking at the individual scientist and the broader scientific and societal implications of the research, not necessarily captured by bibliometric parameters, is the best way to go.

Numerous individual parameters are not considered and not captured in conventional bibliometry-driven rankings. There are striking differences in individual career paths of highly successful pharmacologists. Lastly, there are unexpected cultural differences between closely related sister disciplines such as pharmacology and biochemistry in terms of bibliometry, yielding systematic disadvantages to pharmacology. To the best of our knowledges, such cultural differences have not yet been considered in decision-making.

### Future studies

Similar analyses can be carried out not only for the members of the DGPT, but also for other scientific societies in Germany. This would require a comparison of the membership lists of various scientific societies with the scientist ranking on *Research.com*. It would also be interesting to compare the elite members of the DGPT with the elite members of other leading international pharmacological societies such as the *British Pharmacological Society (BPS)*, the *American Society for Pharmacology and Experimental Therapeutics (ASPET)* and the *Japanese Pharmacological Society (JPS)*. Such comparative analyses would shed light on the cultural differences in pharmacology in various countries with different academic systems. Based on such analyses, advantages and disadvantages of the various systems can be unmasked and measures for improving the systems could be taken.

## Data Availability

All source data for this study are available upon reasonable request. To comply with data protection laws, the ranking of individual scientists in Fig. 14 will not be disclosed. Along the same line, (academic) ages of scientists will not be disclosed.

## Abbreviations

ANOVA: Analysis of variance
C/P-Index: Quotient from citations by publications
DGKliPha: Deutsche Gesellschaft für Klinische Pharmakologie und Therapie (German Society for Clinical Pharmacology)
DGP: Deutsche Gesellschaft für Pharmakologie (German Society for Pharmacology)
DGPT: Deutsche Gesellschaft für Experimentelle und Klinische Pharmakologie und Toxikologie (German Society for Experimental and Clinical Pharmacology and Toxicology)
e.V.: eingetragener Verein (registered association)
Fig.: Figure
GT: Gesellschaft für Toxikologie (Society for Toxicology)
h-Index: Hirsch Index
h/P-Index: Quotient from Hirsch Index by publications
max: Maximum
min: Minimum
ns: Not significant
*p*: *p* value
PPY: Publications per year
PP2Y: Publications per 2 years
SD: Standard deviation
UNESCO: United Nations Educational, Scientific and Cultural Organization
